# Quality of Life and Associated Factors in Young Workers

**DOI:** 10.3390/ijerph18042153

**Published:** 2021-02-23

**Authors:** José Andrade Louzado, Matheus Lopes Cortes, Márcio Galvão Oliveira, Vanessa Moraes Bezerra, Sóstenes Mistro, Danielle Souto de Medeiros, Daniela Arruda Soares, Kelle Oliveira Silva, Clávdia Nicolaevna Kochergin, Vivian Carla Honorato dos Santos de Carvalho, Welma Wildes Amorim, Sotero Serrate Mengue

**Affiliations:** 1 Multidisciplinary Health Institute, Federal University of Bahia, Vitória da Conquista 45029094, Bahia, Brazil; matheuscortes@hotmail.com (M.L.C.); mgalvao@ufba.br (M.G.O.); vanessaenut@yahoo.com.br (V.M.B.); smistro@ufba.br (S.M.); daniellesoutomedeiros@gmail.com (D.S.d.M.); dandani23@yahoo.com.br (D.A.S.); kelle.oliveira@gmail.com (K.O.S.); kochergin62@gmail.com (C.N.K.); vihonorato@hotmail.com (V.C.H.d.S.d.C.); 2 Department of Natural Sciences, State University of Southwestern Bahia, Vitória da Conquista 45083900, Bahia, Brazil; welma.wilde@uesb.edu.br; 3 Graduate Program in Epidemiology, School of Medicine, Federal University of Rio Grande do Sul, Porto Alegre 90035003, Rio Grande do Sul, Brazil; sotero@ufrgs.br

**Keywords:** quality of life, occupational health, categories of workers, motor activity

## Abstract

Background: This study aimed to identify the factors associated with the quality of life of young workers of a Social Work of Industry Unit. Methods: This was a cross-sectional study conducted on 1270 workers. Data were collected using a digital questionnaire built on the KoBoToolbox platform that included the EUROHIS-QOL eight-item index to assess quality of life. Demographic, socioeconomic, behavioral, and clinical variables were considered explanatory. The associations were analyzed using the ordinal logistic regression model at a 5% significance level. Results: Men and women had a mean quality of life of 31.1 and 29.4, respectively. Workers that rated their health as “very good” had an odds ratio of 7.4 (95% confidence interval (CI) = 5.17–10.81), and those who rated it as “good” had an odds ratio of 2.9 (95% CI = 2.31–3.77). Both these groups of workers were more likely to have higher levels of quality of life as compared to workers with “regular”, “poor”, or “very poor” self-rated health. Physically active individuals were 30% more likely to have higher levels of quality of life (odds ratio = 1.3; 95% CI = 1.08–1.65). After adjusting the model by gender, age group, marital status, socioeconomic class, self-rated health, nutritional status, and risky alcohol consumption, the odds ratio of active individuals remained stable (odds ratio = 1.3; 95% CI = 1.05–1.66). Conclusions: In the present study, self-rated health, physical activity, and gender were associated with young workers’ quality of life.

## 1. Background

Quality of life (QoL) is an important measure for evaluating the health situation of individuals and populations and is strongly influenced by the constant environmental, technological, economic, and labor relation changes. In this sense, QoL is an important indicator of individual and collective health, provided that it is measured based on its complex conceptual framework [1,2,3].

Since the establishment of the World Health Organization Quality of Life (WHOQOL) group, the World Health Organization (WHO) expanded the concept of QoL, adding to it the individual’s understanding of their life condition in the context of cultural and social values in response to the expectations and concerns encountered in the development of their life plan [1,2].

Several factors interfere with QoL; these are related to the multidimensionality and subjectivity of its conceptual aspects. The worker’s lifestyle can be a determining factor for health risk behavior, clinical condition, physical activity (PA), and self-rated health. These elements, in turn, potentially affect QoL [1,2,4,5].

In Brazil, the industry and commerce sector accounts for approximately 40% of the country’s formal jobs [6]. Therefore, it is important to measure the QoL levels of formal workers as the results could be used as occupational health indicators, thus contributing to the understanding of health conditions, the construction/implementation of public policies, and the planning of systematized actions for providing care [1,2,3].

QoL levels in workers are affected by their interaction with the labor market, which requires high productivity but offers inadequate working conditions and low pay most of the time. Some workers still live with occupational and chronic-degenerative diseases, which directly interfere with their QoL and promote presenteeism at work [7,8].

Empirical studies on QoL do not address formal workers; most of the data available on the topic refer to specific groups, such as women, the elderly, and people with chronic diseases. Thus, no information about young and healthy workers, which represents the majority of the workforce, is available. Investigating this topic may contribute to reducing the knowledge gap regarding this group’s QoL levels and add to the theoretical and epidemiological framework of public policies on occupational health.

Identifying the QoL levels of workers and recognizing it as a potential indicator of occupational health may allow the development of health promotion actions, which could make the work environment more compatible with an increasing market production without causing occupational pathologies in workers [3,9,10]. Therefore, thias study aimed to identify the factors associated with QoL in young workers from a municipality in Northeast Brazil.

## 2. Methods

### 2.1. Study Design and Population

This was a cross-sectional study conducted in Brazil on users of the HealthRise program—a program targeting the reduction of premature death from chronic non-communicable diseases, such as diabetes and hypertension—in Vitória da Conquista, aimed at improving users’ access to primary health services, qualifying care, implementing medical records, and offering special tests to patients with chronic diseases (hypertension and diabetes) [11].

The municipality of Vitória da Conquista is located in Northeastern Brazil, with an estimated population of 338,000 inhabitants in 2019 and a territorial area exceeding 3,700,000 km^2^ [12]. Most of this population lives in urban areas, which corresponds to approximately 87% [12]. The municipality is located in an important road junction that services the outflow of production between Southeastern and Northeastern Brazil and has a service-based economy with a predominance of the health, education, commerce, and civil construction sectors.

### 2.2. Data Collection

Data were collected at Industry Social Service (SESI), a private, non-profit entity, whose mission is to qualify the workforce and promote workers’ health [13]; thus, the participants were workers assisted by SESI. The inclusion criteria for participants were workers aged 18 years or above, living in the municipality, and having attended SESI for periodic consultations with the occupational doctor. The exclusion criteria were workers coming from other municipalities or those awaiting medical evaluation before dismissal.

Data were collected between August 2017 and July 2018. We sought our study sample from the 3727 workers that scheduled consultations with an occupational physician during this period. However, 339 of them did not attend their consultations, 833 awaited medical evaluation after being dismissed, 516 were from other municipalities, 25 were under 18 years old, and 744 refused to participate in the study, all of which were consequently excluded from the study. Further information about the sample can be found in the “Results” section.

#### 2.2.1. Procedure

Data were collected by trained interviewers (undergraduate health students) using a digital questionnaire built using the KoBoToolbox platform, on tablets. The questionnaire was adapted from the Brazilian National Health Survey 2013 [14] and included information relevant to the outcomes of the project, such as evaluating the self-care of patients with chronic diseases (hypertension and diabetes), evaluating users’ access to health services, and measuring workers’ stress levels and QoL. 

Additionally, the objective measurements of weight and height were collected. A properly calibrated SECA 813^®^ portable digital electronic scale was used for measuring weight, with the participants barefoot and wearing light clothing. To measure height, a portable NutriVida^®^ stadiometer was used, with the participants barefoot and in an upright position.

#### 2.2.2. Instruments and Measurement Variables

QoL was considered the outcome variable and was measured using the EUROHIS-QOL eight-item index, an instrument created by WHO together with the WHOQOL group, aimed at developing research instruments that produce health indicators through an economic approach and have a possible application in different countries to facilitate comparison between Brazilian data and data from other countries [1,2]. 

The EUROHIS-QOL eight-item index instrument was validated and translated into Brazilian Portuguese. It consists of eight questions based on the four WHOQOL-BREF domains: physical health, psychological health, social relationships, and environment, with each item rated on a five-point Likert-type scale ranging from 1 to 5. The total QoL score is obtained by adding the scores on all items, and ranges from 8 to 40, with higher scores indicating higher QoL [1,2]. QoL was treated as a continuous variable and categorized into tertiles.

Demographic, socioeconomic, behavioral, and clinical variables were considered explanatory variables. The Brazil Economic Classification Criterion (*Critério de Classificação Econômica Brasil*—CCEB) of the Brazilian Association of Research Companies (*Associação Brasileira de Empresas de Pesquisa*—ABEP), which came into force from 2015, was used with an update of the class distribution in 2016 [15]. The demographic and socioeconomic variables of interest were gender, age, socioeconomic class, marital status, and work shift. Only two categories were considered for marital status: living or not living with a partner. Work shift was also divided into two levels: those working exclusively during daytime and other work shift modalities.

The behavioral variables considered in this study were diet, smoking, alcohol consumption, and PA. Healthy eating was defined as the consumption of greens, vegetables, fruits, and fruit juices, with at least one portion of fruit or fruit juice and two servings of greens and vegetables at least five times a week [14,16]. Workers, who used tobacco in any quantity, even sporadically, were considered smokers (14). Risky alcohol consumption was described as consuming four or more doses of alcohol for women and five or more doses for men on the same occasion within the last 30 days [14]. PA was assessed using the International Physical Activity Questionnaire (IPAQ), and participants who engaged in more than 150 min of PA per week were considered to be physically active [17,18]. 

The clinical variables included were self-rated health (14) (grouped into three levels: “Very good”, “Good”, and “Regular, Poor, and Very Poor”), and nutritional status, which was classified by body mass index (BMI = weight/height^2^) and grouped into two categories: non-obese workers (BMI ≤ 29.9 kg/m^2^) and obese workers (BMI ≥ 30 kg/m^2^) [19,20,21,22]. 

### 2.3. Statistical Analysis

The descriptive analysis of the study variables was initially performed. Continuous variables were represented by means, and categorical variables were represented in simple frequencies and percentages. The homogeneity of variance of means was evaluated by Levene’s test. ANOVA or Brown–Forsythe tests were used to determine the differences between means, and a Tukey’s HSD test was used to show the differences. Two-way ANOVA was used to compare the mean QoL in men and women, taking into account socioeconomic and clinical characteristics. 

Cumulative odds ordinal logistic regression stepwise format with proportional odds was used to determine the effect of risky behaviors, clinical conditions, and demographic and socioeconomic determinants on QoL levels. A significance level of 20% (*p* < 0.20) was used in the model for the explanatory variables. The proportional odds were evaluated by a total likelihood ratio test, comparing the fitted models with models with variable localization parameters. The adjustment quality deviation test indicated that the model had a good fit and significantly predicted the dependent variable. Logistic regression produced estimates, which were calculated by points and intervals with a 95% confidence and a significance level of 5%. Statistical analyses were performed using IBM SPSS Statistics software version 27 (IBM Corp., Armonk, NY, USA). 

### 2.4. Ethical Considerations

The research was approved by the Research Ethics Committee of the Federal University of Bahia/Multidisciplinary Institute in Health—Anísio Teixeira Campus, according to CAEE number 62259116.0.0000.5556. All the participants signed an informed consent form.

## 3. Results

The mean age of the 1270 workers who participated in the study was 33 years (standard deviation = 10), and most of them were men (80.0%). Of them, 49.5% belonged to social class C and 62.2% were married or lived with a partner. The predominant work shift was the daytime shift (81%). A total of 86.6% reported having good or very good health. The prevalence of obesity was 14.8%, while that of unhealthy eating was 56%. Tobacco use was reported by only 8.4%, while risky alcohol consumption was reported by 28.7%. A total of 62.3% of the workers engaged in PA daily (Table 1).

The bivariate analysis of socioeconomic and clinical conditions showed a statistically significant difference in the QoL between men and women; men had a higher mean QoL (31.1) than women (29.4). The groups with higher mean QoL were the ≤29 years and ≥50 years age groups with QoL scores of 31.0 and 31.2, respectively, followed by social classes A and B with a score of 31.2, and workers who reported very good health with a score of 33.1 (Table 2).

The bivariate analysis of QoL, including risky behavior, habits, and lifestyle, showed statistically significant differences with higher mean QoL for individuals classified as non-obese (30.9), non-smoker (30.8), and physically active (31.0). However, individuals who practiced risky drinking had a higher mean QoL level (31.2) than those who did not (30.6) (Table 2). 

The comparison between the QoL in men and women using two-way ANOVA showed statistically significant differences for all demographic, socioeconomic, behavioral, and clinical variables (Table 3). 

The ordinal logistic regression analysis showed that QoL was likely to be 30% higher in individuals who were physically active (OR = 1.3; 95% CI = 1.08–1.65), and even after the odds ratio was adjusted for gender, age group, marital status, socioeconomic class, self-rated health, nutritional status, and risky alcohol consumption, it remained stable and statistically significant (Model 5) for active individuals (OR = 1.3; 95% CI = 1.05–1.66) (Table 4). 

Males had a higher odds ratio value for QoL than females in all logistic regression models. It is worth noting that after adjusting the model by age group, marital status, and socioeconomic class (Model 3), men were twice as likely to have a higher QoL as women were (Table 4).

Workers who reported having very good (OR = 7.4; 95% CI 5.17–10.81) or good (OR 2.9; 95% CI 2.31–3.77) health (Model 5) were approximately six and seven times more likely to have a higher QoL, respectively, when compared to workers who reported having regular, poor, or very poor health (Table 4). 

Model 1: Effect of physical activity without adjustment. Nagelkerke R² = 0.006

Model 2: Physical activity adjusted by gender, age group, and marital status. Nagelkerke R² = 0.035

Model 3: Physical activity adjusted by gender, age group, marital status, and socioeconomic class. Nagelkerke R² = 0.046

Model 4: Physical activity adjusted by gender, age group, marital status, socioeconomic class, and self-rated health. Nagelkerke R² = 0.168

Model 5: Physical activity adjusted by gender, age group, marital status, socioeconomic class, self-rated health, nutritional status, and risky alcohol consumption. Nagelkerke R² = 0.171

## 4. Discussion

The main factors associated with QoL in young workers in the present study were the male gender and regular PA practice, even in the model adjusted (ordinal logistic regression) for sociodemographic, economic, behavioral, and clinical variables. The stable QoL level in physically active individuals, even in the analysis model, reinforces the positive association of PA with all QoL domains.

The studied population of formal workers comprises young individuals, predominantly males, which is similar to other studies conducted in Brazil, since 56% of formal jobs are occupied by men [23]. Although there is a movement in government and non-governmental institutions in the contemporary world for valuing and inserting women in the labor market, these actions cannot guarantee gender equality in the productive sector.

Most workers in the present sample were married, practiced PA, and reported having good or very good health. Moreover, some studies indicate a positive relationship between PA and self-rated health. This association may be due to the benefits generated by the practice of PA, such as reduced incidence of diseases, improved self-esteem, and cognitive ability, and promotion of social contact with people with healthy habits, which may favor a better self-rating of health conditions, which is a good health indicator for the population [24,25]. 

Self-rated health has good reliability and validity not only as a predictor of morbidity and mortality but also for identifying the health needs of the formal worker population and, more objectively, for stratifying their clinical health conditions and behavioral attitudes [25,26]. Most workers (men and women) who reported having good or very good health also reported higher QoL levels, as identified in a Brazilian population-based study [26] in which self-rated health was 74.2%, a percentage that reduces as the number of morbidities increases, leading to worse self-rated health and reduced Q_O_L. 

The study showed better QoL levels among workers who practiced risky drinking. This result may be partially explained by the fact that the workers in this study were mostly young. Another explanation could be that as alcohol consumption is often associated with moments of leisure and partying, it may subjectively be perceived as spending quality time [25,27]. 

Gender is an important variable when we consider the historically consolidated differences between men and women, and the same was found in the results of this study. Men had a higher quality of life and occupied more job positions than women did. Several studies on the role of gender in health present possible causes for these differences, including double working hours for women (employment and home activities), number of children, and the difficulty of entering the labor market faced by women [10,23]. 

In this sense, the power relations established by a gender bias have historically built the social division of labor, based on biological aspects associated with sexist social stereotypes and cultural norms of appreciation of men, consequently limiting women to unpaid activities and jobs considered to be of little administrative and economic relevance. This trend continues today, as can be seen in the income gap by gender, such that in 2019, Brazilian women had a mean income 11.63% lower than that of men [6,28,29]. The inclusion of women in precarious job positions with low pay has a negative impact on QoL, as income is one of the determinants of lifestyle, access to goods and services, and, consequently, health condition, which is influenced by working life [28,30]. 

Physically active individuals showed higher QoL levels than those who were inactive. We constructed a model in which the main variables of the database were used to better understand their relationship with the outcome of the study (QoL). The main exposure variable in the model was PA, as the available literature shows that there is a relationship between PA and QoL level. The model derived from ordinal logistic regression showed a certain stability of QoL levels in physically active individuals [5,31,32]. This is because the benefits of PA go beyond the improvement of the clinical and biological condition since it promotes social interaction, the establishment of bonds of friendship, and emotional balance, which are subjective and integral elements of the multidimensional aspect of the QoL construct [32]. 

Our findings support that PA is a variable that directly and indirectly positively influences all domains of QoL (physical, psychological, social, environmental, and general health condition) [1]. The results of studies show that PA induces behavioral change, which is fundamental for disease control and prevention. Thus, the incorporation of PA in daily life becomes an important therapeutic alternative, capable of improving general health conditions, which necessarily results in better QoL levels for workers [31,32].

Most workers were considered active. This may be due to the benefits of PA practice, the desire for a better body image, and the availability of public equipment (bike paths, hiking tracks, and fitness equipment in squares and health units). Moreover, the population mostly comprised young people [33]. However, a significant proportion of workers were inactive; this may be a reflection of technological evolution that is providing comfort, increased productivity, reduction of time requirements and work, and at the same time, less physical effort.

No statistically significant differences in QoL were found in terms of age and marital status in this study. Although age is an important factor in the labor market, it was not present in the regression and was associated with increased QoL levels. These results differ from those found in the literature [10]. However, when age is analyzed separately, workers over 50 years of age had the highest QoL means, not agreeing with the findings of other studies [9,10]. This contrast may be due to the greater financial resources and professional stability of these workers [34].

The higher socioeconomic classes (A, B1, and B2) had higher incomes, and consequently, greater purchasing power of goods and services, stability in work relationships, and more job satisfaction, which are determining factors for physical and mental health, essential elements for QoL [15,26,35]. Their economic and social position seems to be a determining factor of QoL levels, which is consistent with the results of our study: workers of higher socioeconomic classes had better QoL in all proposed models [26,35].

Self-rated health is influenced by subjective and objective criteria, and according to previous studies, it is a good predictor of mortality, being a reflection of biological, socioeconomic, and behavioral aspects [36]. We found that individuals who reported having better health had higher QoL levels. Nutritional status and alcohol consumption, in turn, were not associated with QoL since the results did not show statistical significance [25]. 

The findings of this study should be interpreted considering some methodological limitations. First, the presence of acute pathologies was not assessed when data were collected, which may have influenced QoL levels. Second, in terms of the study design, as cross-sectional studies are limited to identifying associations and causal relationships cannot be established, reverse causality can occur [37]. For example, although lower levels of QoL were associated with obesity, it can be argued that the problems caused by overweight (chronic diseases, emotional damage, and functional limitations) obstruct QoL.

The results of this study support the use of QoL as an epidemiological indicator for the planning of health-related arrangements for workers. Because QoL is an indicator of health conditions, it can be adopted into all levels of the healthcare system, especially primary care, which is usually a worker’s first point of contact with the health system, to identify general health needs and diagnose possible occupational diseases. 

## 5. Conclusions

Most of the participant workers engaged in regular PA, and demographic, socioeconomic, clinical, and some behavioral factors had little influence on QoL levels. The results of this study may contribute to guiding the implementation of public policies that systematically promote PA in the daily lives of workers. It also highlights the need for further studies to investigate factors associated with QoL in women to confirm and possibly deepen the understanding of the present results.

## Figures and Tables

**Table 1:** Demographic, socioeconomic, clinical, and behavioral aspects of workers.

Variable	*n*	%
Gender Male	1019	80.0
Female	251	20.0
Age Group Up to 29 years	469	36.9
30–39 years	492	38.8
40–49 years	217	17.1
50 years or above	92	7.2
Socioeconomic Class ** A + B1 + B2	465	36.6
C1 + C2	629	49.5
D + E	176	13.9
Marital Status * Married or Living Together	789	62.2
Single/Divorced/Widowed	480	37.8
Work Shift * Daytime	1014	81.0
Nighttime/Daytime and Nighttime/Nighttime on duty	238	19.0
Self-Rated Health * Very good	170	13.4
Good	645	50.8
Regular/Poor/Very poor	454	35.8
Nutritional Status * Non-obese	1051	85.2
Obese	183	14.8
Eating * Healthy eating	523	44.0
Unhealthy eating	665	56.0
Tobacco Use * Yes	107	8.4
No	1162	91.6
Risky Alcohol Consumption Yes	364	28.7
No	906	71.3
Physical Activity Active	791	62.3
Non-active	479	37.7
Quality of Life * Up to 30	532	42.1
31–32	347	27.4
33 or more	386	30.5

* Variables with missing data; ** These criteria evaluate individuals’ socioeconomic level through a household assessment. Scores range from 0 to 100 points, with higher scores representing a higher economic stratum: A (45–100 points), B1 (38–44 points), B2 (29–37 points), C1 (23–28 points), C2 (17–22 points), and D/E (≤16 points).

**Table 2:** Quality of life and demographic, socioeconomic, clinical, and behavioral characteristics of workers.

Variable	Quality of Life	P
Gender Male	31.11	0.000 *^3^
Female	29.44	
Age Group Up to 29 years	31.05	0.011 *^2^
30–39 years	30.71	
40–49 years	30.13	
50 years or above	31.28	
Socioeconomic Class *⁴ A + B1 + B2	31.26	0.002 *^2^
C1 + C2	30.55	
D + E	30.33	
Marital Status Married or Living Together	30.79	0.086 *^1^
Single/Divorced/Widowed	30.76	
Work Shift Daytime	30.81	0.652 *^1^
Nighttime/Daytime and Nighttime/Nighttime on duty	30.68	
Self-Rated Health Very good	33.18	0.000*^3^
Good	31.50	
Regular/Poor/Very poor	28.88	
Nutritional Status Non-obese	30.99	0.000 *^1^
Obese	29.63	
Eating Healthy eating	30.91	0.258 *^1^
Unhealthy eating	30.66	
Tobacco Use Yes	30.44	0.329 *^1^
No	30.81	
Risky Alcohol Consumption Yes	31.23	0.000 *^1^
No	30.60	
Physical Activity Active	31.00	0.006 *^1^
Non-active	30.41	

*^1^ Student’s *t*-test; *^2^ ANOVA; *^3^ Brown–Forsythe; *⁴ These criteria evaluate individuals’ socioeconomic level through a household assessment. Scores range from 0 to 100 points, with higher scores representing a higher economic stratum: A (45–100 points), B1 (38–44 points), B2 (29–37 points), C1 (23–28 points), C2 (17–22 points), and D/E (≤16 points).

**Table 3:** Quality of life and demographic, socioeconomic, and clinical characteristics of working men and women.

Variable	Size Effect	Quality of Life		P
		Men	Women	
Age Group Up to 29 years	0.009	31.33	29.92	Gender = 0.000 Age = 0.100 Gender * Age = 0.390
30–39 years		31.09	29.26	
40–49 years		30.41	29.15	
50 years or above		31.60	28.00	
Socioeconomic Class ** A + B1 + B2	0.010	31.77	29.82	Gender = 0.000 SC = 0.004 Gender * SC = 0.861
C1 + C2		30.83	29.12	
D + E		30.57	29.00	
Marital Status Married or Living Together	0.000	31.08	29.28	Gender = 0.000 MS = 0.433 Gender * MS = 0.622
Single/Divorced/Widowed		31.16	29.61	
Work Shift Daytime	0.000	31.18	29.45	Gender = 0.000 WS = 0.493 Gender * WS= 0.952
Night/Daytime and Night/Nighttime on duty		30.89	29.21	
Self-Rated Health Very good	0.171	33.46	32.03	Gender = 0.000 SRH = 0.000 Gender * SRH = 0.050
Good		31.63	30.83	
Regular/Poor/Very Poor		29.38	28.88	
Nutritional Status Non-obese	0.017	31.23	29.92	Gender = 0.000 NS = 0.000 Gender * NS = 0.011
Obese		30.40	27.35	
Eating Healthy eating	0.001	31.37	29.19	Gender = 0.000 Eating = 0.926 Gender * Eating = 0.060
Unhealthy eating		30.90	29.71	
Tobacco Use Yes	0.001	30.57	27.25	Gender = 0.008 Tobacco use = 0.131 Gender * Tobacco use = 0.385
No		31.17	29.48	
Risky Alcohol Consumption Yes	0.006	31.48	29.16	Gender = 0.000 Alcoholism = 0.764 Gender * Alcoholism = 0.200
No		30.93	29.50	
Physical Activity Active	0.006	31.38	29.42	Gender = 0.000 PA = 0.208 Gender * PA = 0.137
Non-active		30.66	29.48	

* Two-way ANOVA; ** These criteria evaluate individuals’ socioeconomic level through a household assessment. Scores range from 0 to 100 points, with higher scores representing a higher economic stratum: A (45–100 points), B1 (38–44 points), B2 (29–37 points), C1 (23–28 points), C2 (17–22 points), and D/E (≤ 16 points).

**Table 4:** Association between quality of life and demographic, socioeconomic, clinical, and behavioral variables.

Variable	Β	Model 1 OR (95% CI)	Β	Model 2 OR (95% CI)	Β	Model 3 OR (95% CI)	Β	Model 4 OR (95% CI)	Β	Model 5 OR (95% CI)
Physical Activity Non-active		1		1		1		1		1
Active	0.288	1.33 (1.08–1.65)	0.262	1.30 (1.05–1.61)	0.275	1.31 (1.06–1.63)	0.271	1.31 (1.05–1.64)	0.279	1.32 (1.05–1.66)
Gender Female				1		1		1		1
Male			0.634	1.88 (1.44–2.47)	0.702	2.02 (1.54–2.65)	0.633	1.88 (1.41–2.49)	0.611	1.84 (1.37–2.48)
Age Group 50 years or above				1		1		1		1
Up to 29 years			−0.043	0.95 (0.63–1.47)	0.053	1.05 (0.68–1.62)	−0.054	0.94 (0.61–1.48)	−0.071	0.93 (0.59–1.46)
30–39 years			−0.132	0.88 (0.58–1.32)	−0.053	0.94 (0.62–1.44)	−0.103	0.90 (0.59–1.39)	−0.121	0.89 (0.57–1.37)
40–49 years			−0.494	0.61 (0.39–0.96)	−0.393	0.68 (0.42–1.07)	−0.361	0.69 (0.43–1.12)	−0.372	0.69 (0.43–1.11)
Marital Status Single/Divorced/Widowed				1		1		1		1
Married or Living Together			0.095	1.09 (0.88–1.38)	0.118	1.12 (0.89–1.41)	0.146	1.16 (0.91–1.47)	0.178	1.19 (0.93–1.52)
Socioeconomic Class * D + E A + B1 + B2 C1 + C2					0.562 0.168	1 1.75 (1.27–2.42) 1.18 (0.88–1.59)	0.509 0.223	1 1.66 (1.19–2.31) 1.25 (0.92–1.69)	0.525 0.244	1 1.69 (1.20–2.38) 1.28 (0.94–1.74)
Self-Rated Health Regular/Poor/Very poor								1		1
Very good							2.039	7.68 (5.37–11.00)	2.012	7.47 (5.17–10.81)
Good							1.094	2.98 (2.34–3.79)	1.083	2.95 (2.31–3.77)
Nutritional Status Obese Non-obese									0.228	1 1.26 (0.91–1.72)
Risky Alcohol Consumption No										1
Yes									0.152	1.16 (0.91–1.48)

OR: odds ratio; CI: confidence interval; * These criteria evaluate individuals’ socioeconomic level through a household assessment. Scores range from 0 to 100 points, with higher scores representing a higher economic stratum: A (45–100 points), B1 (38–44 points), B2 (29–37 points), C1 (23–28 points), C2 (17–22 points), and D/E (≤16 points).

## Abbreviations

QoL: Quality of life; WHOQOL: World Health Organization Quality of Life; WHO: World Health Organization; PA: Physical activity; SESI: *Serviço Social da Indústria* (Social Work of Industry Unit); CCEB: *Critério de Classificação Econômica Brasil* (Brazil Economic Classification Criterion); ABEP: *Associação Brasileira de Empresas de Pesquisa* (Brazilian Association of Research Companies); IPAQ: International Physical Activity Questionnaire; BMI: Body mass index.
